# CCR2-dependent monocytes gut-homing contributes to enteric neuroplastic changes in a mouse model of diet-induced obesity

**DOI:** 10.1186/s10020-026-01560-y

**Published:** 2026-07-15

**Authors:** Clarissa Pierucci, Sofia Faggin, Laura Benvenuti, Valentina Caputi, Chiara Ippolito, Cristina Segnani, Vanessa D’Antongiovanni, Giulia Valdiserra, Lorenzo Flori, Rocchina Colucci, Maria Cecilia Giron, Luca Antonioli, Nunzia Bernardini, Carolina Pellegrini

**Affiliations:** 1 Unit of Histology and Medical Embryology, Department of Clinical and Experimental Medicine, University of Pisa, Pisa, Italy; 2 Department of Pharmaceutical and Pharmacological Sciences, University of Padova, Largo E. Meneghetti, Padua, 2 - 35131 Italy; 3 Institute of Digestive Health Research (IRSD), Toulouse University, INSERM U1022, INRAe, ENVT, Toulouse, France; 4 Department of Translational Research and New Medical and Surgical Technologies, University of Pisa, Pisa, Italy; 5 Department of Poultry Science, University of Arkansas, Fayetteville, AR 72701 USA; 6 Unit of Pharmacology and Pharmacovigilance, Department of Clinical and Experimental Medicine, University of Pisa, Pisa, Italy; 7 Department of Pharmacy, University of Pisa, Pisa, Italy

**Keywords:** Colonic motility, High fat diet, Obesity, Substance P, Tachykininergic neurotransmission, *Muscularis* macrophages, CCL2/CCR2 pathway, Monocyte gut-homing

## Abstract

**Background:**

Enteric inflammation and immune cell activation promote enteric neuroplastic changes and CCR2-dependent monocyte recruitment, contributing to obesity-associated colonic dysmotility and chronic inflammation. Here, we examined the role of C–C motif chemokine ligand (CCL2)/C–C chemokine receptor 2 (CCR2)-mediated blood monocytes gut-homing in colonic motor dysfunctions in mice with high fat diet (HFD)-induced obesity.

**Methods:**

Wild-type C57BL/6J mice were fed HFD or standard diet (SD) for 8 weeks. Subgroups received the CCR2 antagonist RS504393 (2 mg/kg/day, oral) or vehicle throughout the HFD intake. Body weight and body mass index (BMI) were monitored starting from HFD intake. Colonic transit was assessed by fecal pellet output. In vitro colonic contractility was evaluated by assessing substance P (SP)-induced contractile responses, along with analysis of SP-positive neurons. Immunohistochemistry was used to quantify CCR2⁺ monocytes and CD64⁺/Iba-1⁺ macrophages within the myenteric plexus. Colonic expression of CCL2 in enteric neurons (HuC/D⁺) and glial cells (GFAP⁺) was assessed. Gut vascular barrier integrity was evaluated via PV-1 expression and plasma lipopolysaccharide-binding protein (LBP) levels. The effects of CCR2 blockade on motility, neuronal SP expression, chemokine expression, and monocyte recruitment were analyzed.

**Results:**

HFD mice displayed an increase in body weight and BMI. An altered in vivo colonic transit along with an increased in vitro tachykininergic contractions and myenteric SP immunopositivity were detected in HFD mice. HFD mice exhibited marked infiltration of monocyte-derived macrophages (CD64⁺, Iba-1⁺) within the muscularis layer, in close spatial association with SP-positive nerve fibers and neuromuscular vasculature. Increased CCL2 expression was observed in both enteric neurons and glial cells, alongside evidence of compromised gut vascular barrier function. Pharmacological CCR2 inhibition attenuated weight gain and significantly improved colonic transit. Moreover, CCR2 blockade normalized tachykininergic hyperactivity, reduced SP expression, decreased colonic CCL2 levels, and limited monocyte/macrophage accumulation in the enteric neuromuscular compartment.

**Conclusion:**

During obesity, the activation of the CCL2/CCR2 pathway promotes the recruitment of blood-derived monocytes to the gut, driving neuroimmune remodelling within the enteric nervous system. Blood monocyte-derived macrophages modulate tachykininergic pathways, contributing to colonic dysmotility. Targeting CCR2-dependent gut immune cell trafficking may represent a novel therapeutic strategy for obesity-associated GI dysfunction.

**Graphical Abstract:**

**Figure:**
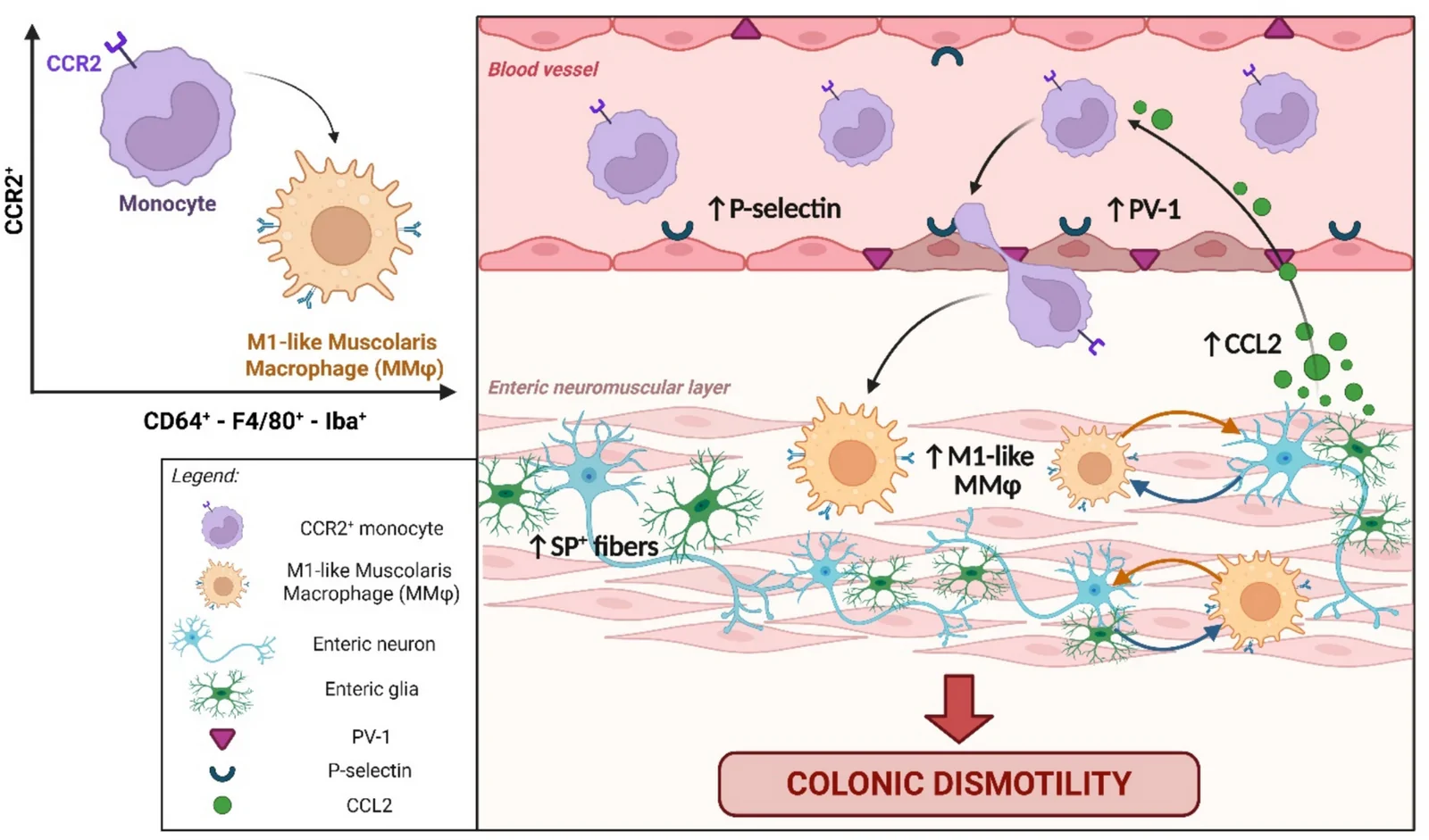

**Supplementary Information:**

The online version contains supplementary material available at https://doi.org/10.1186/s10020-026-01560-y.

## Background

Accumulating evidence suggests that immune-metabolic imbalances contribute to the pathophysiology of obesity and related comorbidities, including cardiovascular diseases, gastrointestinal (GI) dysfunctions, nonalcoholic steatohepatitis (NASH), and cognitive impairment (Kloock et al. 2023; Le Pluart et al. 2015). In particular, gut dysbiosis and impairment of intestinal barrier following high-fat diet (HFD) assumption promote the translocation of luminal pathogens into the intestinal mucosa, triggering enteric immune responses that, in turn, sustain chronic low-grade inflammation (Antonioli et al. 2020; D’Antongiovanni et al. 2023; Massier et al. 2021). Concurrently, persistent enteric inflammation may promote CCR2-dependent recruitment of circulating monocytes into intestinal tissues, thereby contributing to the progression of chronic local inflammatory responses (Mowat and Agace 2014; Rohm et al. 2021). Of note, obesity-related enteric inflammation has also been linked to enteric neuroplastic changes, that contribute to bowel motor dysfunctions frequently observed in obese patients (Pellegrini et al. 2021). Supporting this view, a previous study demonstrated that depletion of peritoneal macrophages attenuated enteric inflammation and reversed morpho-functional alterations of tachykininergic pathways in obese mice, resulting in normalization of colonic motility (Antonioli et al. 2019). In this context, intestinal muscularis macrophages—located between the serosa, longitudinal muscle, circular muscle, and myenteric neurons – besides regulating intestinal motility in physiological conditions, have been found to contribute to inflammation-driven dysmotility through the release of inflammatory mediators and recruitment of additional immune/inflammatory cells (Muller et al. 2014; Correction: CCR2-dependent monocyte-derived macrophages resolve inflammation and restore gut motility in postoperative ileus 2019). However, whether circulating blood-derived monocytes, beyond infiltrating the intestinal mucosa, also accumulate within the enteric neuromuscular compartment polarizing towards a pro-inflammatory macrophage phenotype during obesity remains unknown.

Based on this background, the present study aimed to investigate the role of gut-homing monocytes in obesity-associated bowel dysmotility, with the ultimate goal of identifying novel pharmacological targets for therapeutic intervention.

## Methods

### Animal model of diet-induced obesity

**Fig. 1:**
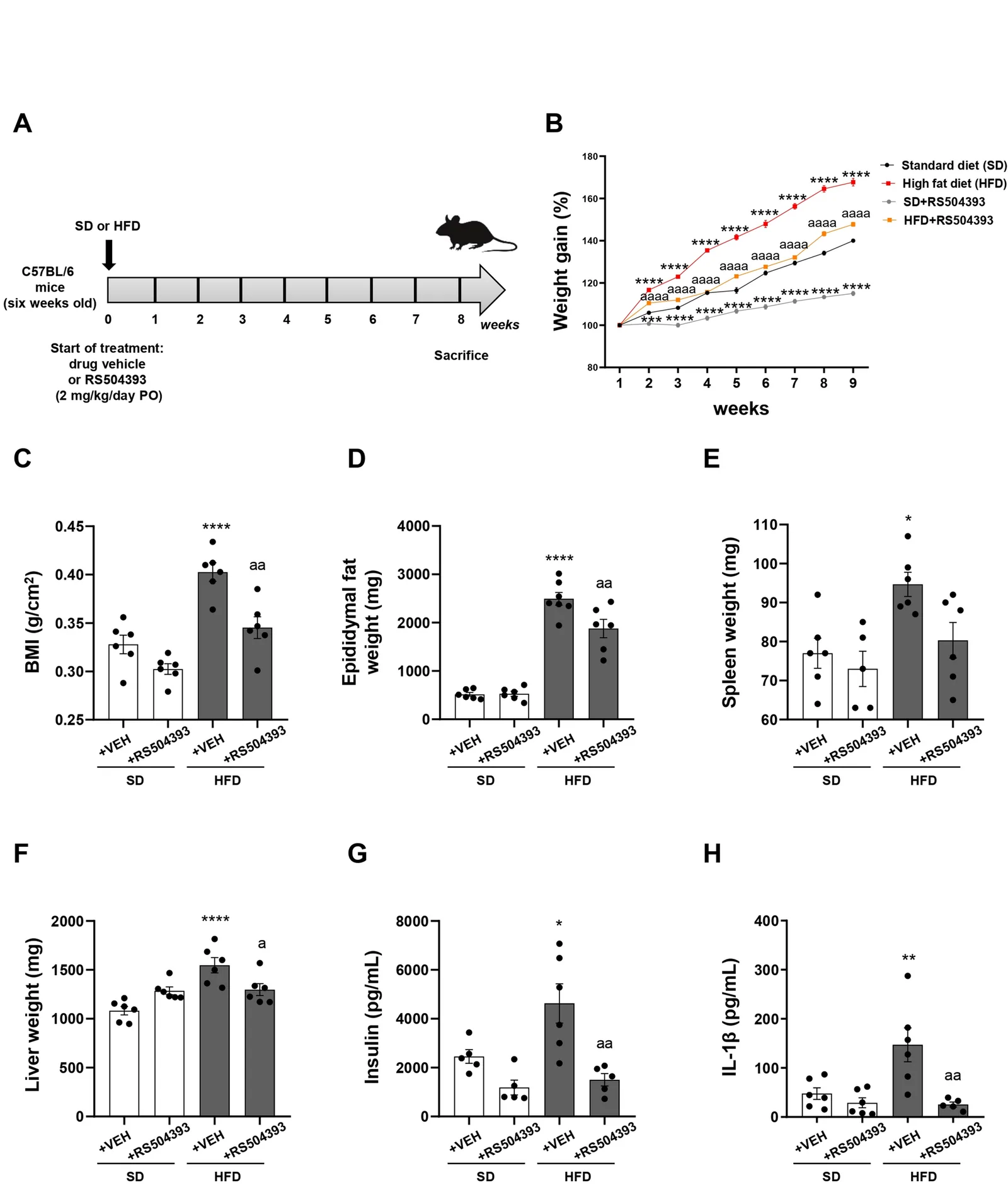
CCR2/CCL2 pathway contributes to the development of HFD-induced obesity. **A** Schematic representation of in vivo treatment of SD and HFD mice (six weeks of age) with vehicle or CCR2 antagonist (RS504393, 2 mg/kg/day orally) for eight weeks. **B**-**G** Effect of RS504393 treatment in HFD mice on metabolic parameters. **B** Body weight gain, **C** body mass index (BMI), **D** epididymal fat weight, **E** spleen weight and **F** liver weight in SD and HFD mice treated with vehicle or RS504393 (*N* = 6 mice/group). **G** Insulin levels in plasma of SD and HFD mice treated with vehicle or RS504393. **H** IL-1β levels in colonic tissues of SD and HFD mice treated with vehicle or RS504393. Data are expressed as mean ± S.E.M. (*N* = 6 mice/group). One-way ANOVA or two-way ANOVA followed by Tukey post hoc test results: *p < 0.05, **p < 0.01, ***p < 0.001, ****p < 0.0001vs. vehicle-treated SD; ^a^*P* < 0.05, ^aa^*P* < 0.01, ^aaa^*P* < 0.001, ^aaaa^*P* < 0.0001 vs. vehicle-treated HFD. *Abbreviations:* SD, standard diet; HFD, high-fat diet

Six-week-old C57BL/6 male mice (20–25 g body weight) were employed throughout the study. The animals were provided by Envigo S.r.l (San Pietro al Natisone UD, Italy) and upon arrival were housed four in a cage in temperature-controlled rooms on a 12 h light cycle at 22–24 °C and 50%–60% humidity, with water and food availability ad libitum. To induce obesity, mice was fed with HFD (high fat diet) for eight weeks. The HFD (Altromin International, Germany; C 1090–60) provided 5.1 kcal/g, with 60.8% kcal as fats, 18.3% kcal as proteins, and 21.4% kcal as carbohydrates, while standard diet (Altromin International, Germany; SD, C 1090–10) provided 3.1 kcal/g, with 18% kcal as fats, 24% kcal as proteins, and 58% kcal as carbohydrates. Animals were randomly divided into four groups, each composed by 6 mice, as follow: SD or HFD plus vehicle for 8 weeks, SD or HFD plus CCR2 antagonist for 8 weeks. CCR2 antagonist (RS504393) was administered via oral gavage, at the dose of 2 mg/kg/day. At the end of the study, the animals were anaesthetized and euthanized (Fig. 1A). Of note, after 8 weeks of HFD, mice are characterized by metabolic alterations such as increase in body and epidydimal fat weight and cholesterol, glucose and triglycerides levels, while no signs of insulin resistance are detected. These conditions closely mirror those observed in obese patients not yet diabetic (Antonioli et al. 2019; Raybould 2012; Csoka et al. 2017). The experiments were approved by the University of Pisa’s Ethical Committee for Animal Experimentation and the Italian Ministry of Health and were carried out following the European guidelines for the handling and use of experimental animals in accordance with the Code of Ethics of the World Medical Association (Declaration of Helsinki, EU Directive 2010/63/EU for animal experiments). Animal studies are reported in compliance with the ARRIVE guidelines and all efforts to reduce and minimize the number of animals, and their suffering were carried out (Kilkenny et al. 2010). The researchers responsible for data analysis were blind to which animals represent SD and HFD mice. A randomization of animals between groups was carried out in order to generate groups of equal size. The design in this study complies with the recommendations on experimental design in pharmacology (Curtis et al. 2018).

### Measurement of body weight, epididymal fat weight and energy intake

Body weight of SD and HFD animals was assessed once a week. During this measurement, all mice were caged individually. At the time of sacrifice epididymal fat, spleen and liver were quickly dissected out and weighed. Body mass index (BMI) was calculated by dividing body weight (g) by body length (mm) squared (BMI = body weight/body length^2^) as described by Smemo et al. (2014).

### Evaluation of faecal output

Faecal output was recorded from 9:00 AM to 10:00 AM the day before the sacrifice. Each animal was removed from its home cage, placed in a clean plastic cage and deprived of food and water for 1 h. Stools were collected immediately after expulsion, counted, weighted and placed in sealed tubes (Pellegrini et al. 2020).

### Recording of contractile activity

The contractile activity of colonic longitudinal smooth muscle preparations was recorded as described previously (Antonioli et al. 2011, 2017; D’Antongiovanni et al. 2020b). After the sacrifice, the colon was removed and put in Krebs solution at 37 °C. Colon samples were opened along the mesenteric border and cut along the longitudinal axis into strips (approximately 3 mm in width and 10 mm in length). The strips were then placed in organ baths containing Krebs solution maintained at 37 °C and continuously bubbled with 95% O₂ and 5% CO₂. Krebs solution had the following composition (in millimolar): NaCl 113, KCl 4.7, CaCl₂ 2.5, KH₂PO₄ 1.2, MgSO₄ 1.2, NaHCO₃ 25, and glucose 11.5 (pH 7.4 ± 0.1). Tissue strips were connected to isometric force transducers (2Biological Instruments, Besozzo, VA, Italy). A tension of 0.5 g was slowly applied to each preparation and mechanical activity was recorded by BIOPAC MP150 (2Biological Instruments). Colonic tissue was allowed to equilibrate for at least 30 min and a pair of coaxial platinum electrodes was positioned at a distance of 10 mm from the longitudinal axis of each preparation to deliver transmural electrical stimulation by a BM-ST6 stimulator (Biomedica M + 07angoni, Pisa, Italy). Electrical stimuli were applied as 10-s trains consisting of square-wave pulses (0.5 ms pulse width, 30 mA amplitude) delivered at a frequency of 10 Hz. After the equilibration period, each preparation was repeatedly challenged with electrical stimulation trains at 5-min intervals, and experiments were started once reproducible responses were obtained (typically after two or three stimulations). Based on the wet tissue weight, the tension developed by each strips, expressed as grams per gram of wet tissue (g/g tissue), was normalized. Before weighing, tissue strips were gently blotted on filter paper to remove excess buffer. Based on previous experiments, the appropriate ES frequency and suitable concentration of exogenous substance P were selected (Pellegrini et al. 2021). In the first series of experiments, electrically induced contractions were recorded while colonic preparations were maintained in standard Krebs solution. In the second set of experiments colonic tissues were maintained in Krebs solution containing N-ω-nitro-L-arginine methylester (L-NAME, nitric oxide synthase inhibitor, 100 μM), guanethidine (adrenergic blocker 10 μM), atropine sulphate (muscarinic receptor antagonist, 1 μM), 5-fluoro-3-[2-[4-methoxy-4-[[(R)-phenylsulphinyl]methyl]-1-piperidinyl]ethyl]-1H-indole (GR159897, NK_2_ receptor antagonist, 1 μM) and (R)-[[(2-phenyl-4-quinolinyl)carbonyl]amino]-methyl ester benzeneacetic acid (SB218795, NK_3_ receptor antagonist, 1 μM) in order to examine the patterns of colonic excitatory motor contractions driven by the tachykininergic NK1 receptors pathway.

In the third series, colonic contractions were evoked by direct pharmacological activation of NK1 receptors located on smooth muscle cells. For this purpose, colonic preparations were maintained in Krebs solution containing tetrodotoxin (TTX, 1 μM) and stimulated with exogenous substance P (1 μM). In addition, longitudinal smooth muscle preparations of distal colon maintained in Krebs solution containing L-NAME (100 μM), guanethidine (10 μM), atropine (1 μM), GR159897 (NK_2_ receptor antagonists, 1 μM), and SB218795 (NK_3_ receptor antagonists, 1 μM) were stimulated with or without L-732,138 (NK_1_ receptor antagonist, 10 μM) or tetrodotoxin (TTX, 1 μM), in order to assess the recruitment of postganglionic tachykininergic motor neurons.

### Evaluation of colonic CCL2, IL-1β, and plasma LBP and insulin levels

Colonic CCL2 and IL-1β levels were measured using commercial ELISA kits (Abcam, Cambridge, United Kingdom; CCL2: ab100722; IL-1β: ab100705), as previously described (Pellegrini et al. 2016). Briefly, colonic tissues previously stored at − 80 °C were thawed, weighed (20 mg), homogenized in PBS (0.4 mL/20 mg tissue) at 4 °C, and centrifuged at 10,000 × g for 5 min. Aliquots (100 μL) of supernatants were then used for the assays. Tissue CCL2 and IL-1β levels were expressed as pg/mL.

Plasma LBP and insulin levels were measured using mouse ELISA kits (LBP: ab213876, Abcam, Cambridge; insulin: CRY-90080, Vinci-Biochem), as previously described (Flori et al. 2026). Blood samples were centrifuged at 4,000 × g for 5 min at 2–8 °C, and aliquots (5 μL) of plasma were used for the assay. Plasma LBP and Insulin levels were expressed as pg/mL.

### Western blot

Colonic tissues were lysed as previously reported (Caputi et al. 2017). Briefly, samples were weighed and homogenized in lysis buffer using a Polytron homogenizer (QIAGEN, Milan, Italy). Total protein concentration was quantified by the Bradford assay. Equal amounts of protein (30 μg) were resolved on pre-cast 4–20% polyacrylamide gels (Mini-PROTEAN® TGX gel, Bio-Rad) and transferred onto PVDF membranes (Trans-Blot® Turbo™ PVDF Transfer Packs, Bio-Rad). Membranes were blocked with 3% BSA prepared in Tris-buffered saline (TBS; 20 mM Tris–HCl, pH 7.5, 150 mM NaCl) supplemented with 0.1% Tween-20. Membranes were then incubated with primary antibodies against actin (1:5000, A3854, Sigma Aldrich) and PV-1 (1:1000, BD Biosciences). HRP-conjugated secondary antibodies were obtained from Abcam (anti-mouse ab97040; anti-rabbit ab6721) and Cell Signaling Technology (anti-rat #7077). Protein bands were detected by enhanced chemiluminescence using Clarity Western ECL Blotting Substrate (Bio-Rad), and densitometric quantification was performed with iBright Analysis Software.

### Confocal immunofluorescence on colonic paraffin sections

Sections (7 μm) from formalin-fixed/paraffin-embedded distal colonic sample sections were processed for single/double/triple immunofluorescence analysis. For selected antibody combinations (CCL2 with HuC/D or GFAP) adjacent serial sections were used to allow analysis of the same anatomical region across distinct markers. In brief, slides were incubated with Protein Block Serum Free and the following primary antibodies: anti-CCL2 with anti-HuC/D or with anti-GFAP, anti-Selectin P (CD62P) or plasmalemmal vesicle associated protein-1 (PV-1) with anti-CD31 or anti-SP with CD31 and anti-Iba-1 diluted in PBS overnight at 4 °C (see Table 1). Successively, samples were treated with appropriate fluorophore-conjugated secondary antibody or biotinylated secondary antibody plus fluorophore-conjugated streptavidin and were finally counterstained with TO-PRO-3 (see Table 1). Sections were examined under a Leica TCS SP8 confocal laser-scanning microscope (Leica Microsystems, Mannheim, Germany), and all images were acquired using XYZ scan mode, HC PL APO CS2 40x/1,30 and HC PL APO CS2 63x/1,40 oil lenses. The acquisition setup included the use of the 488, 555 and 561 laser lines, hybrid detector (HyD) and a gain range of 50/100. Furthermore, Leica Application Suite (L.A.S.) X software allows tri-dimensional (3D) acquisition by sequential scan procedure, zeta stack and 3D viewer function. Representative images of all fields obtained were exported in 8-bit format and with TIFF extension.

**Table 1:** Primary and secondary antibodies and reagents employed in the study

Primary antibodies	Clone	Species	Dilution	Source	Markers
anti- CD62P	MAb	rabbit	1:200	Abcam, Cambridge, UK	Activated endothelial cells
anti-PV-1	MAb	rat	1:600	BD, Biosciences	Vascular permeability
anti-CD31	MAb	rabbit	1:4000	Abcam, Cambridge, UK	Endothelial cells
anti-CD31	MAb	rat	1:50	Dianova a BIOZOL Brand, Hamburg, Germany	Endothelial cells
anti-SP	PAb	rabbit	1:100	Merck SA, Consociata di Merck KGaA, Darmstadt, Germany	SP neurons and fibers
anti-Iba-1	PAb	goat	1:100	Abcam, Cambridge, UK	Activated macrophages
anti-CCL2	Mab	rabbit	1:100	Abcam, Cambridge, UK	C–C Motif Chemokine Ligand 2
anti-GFAP	Mab	rat	1.75	Invitrogen, Eugene, Oregon, USA	Glial cells
anti-HuC/D	MAb	mouse	1:50	Invitrogen, Eugene, Oregon, USA	Neurons
anti-SP	PAb	guinea pig	1:100	Synaptic Systems GmbH, Goettingen, Germany	Positive SP neurons and respective fibers
anti-Iba-1	PAb	rabbit	1:400	FUJIFILM Wako Pure Chemical Corporation	Activated macrophages
anti-CD64	MAb	rabbit	1:50	Invitrogen, Eugene, Oregon, USA	Derived-monocytes activated macrophages
anti-F4/80	MAb	rat	1:50	BioLegend, Amsterdam, Netherlands	Resident and infiltrating macrophages
anti-HuC/D	Mab	mouse	1:100	Invitrogen, Eugene, Oregon, USA	Neurons
Secondary antibodies and reagents					
Streptavidin Alexa Fluor 633 conjugate			1:1000	Invitrogen, Eugene, Oregon, USA	
anti-guinea pig IgG Alexa Fluor 555 conjugate		goat	1:1000	Invitrogen, Eugene, Oregon, USA	
anti-rat IgG Alexa Fluor 488 conjugate		donkey	1:1000	Invitrogen, Eugene, Oregon, USA	
anti-rabbit IgG Alexa Fluor 555 conjugate		goat	1:1000	Invitrogen, Eugene, Oregon, USA	
anti-rabbit IgG DyLight 650		goat	1:1000	Invitrogen, Eugene, Oregon, USA	
Streptavidin, Alexa Fluor^R^ 555 conjugated			1:300	Invitrogen, Eugene, Oregon, USA	
anti-Mouse IgG (H + L) Highly Cross-Adsorbed Secondary Antibody, Alexa Fluor™ 488		donkey	1:300	Invitrogen, Eugene, Oregon, USA	
anti-Rat IgG (H + L) Highly Cross-Adsorbed Secondary Antibody, Alexa Fluor™ Plus 555		donkey	1:300	Invitrogen, Eugene, Oregon, USA	
anti-Rabbit IgG (H + L) Highly Cross-Adsorbed Secondary Antibody, Alexa Fluor™ Plus 647		donkey	1:300	Invitrogen, Eugene, Oregon, USA	
anti-Rabbit IgG (H + L) Highly Cross-Adsorbed Secondary Antibody, Alexa Fluor™ Plus 488		donkey	1:300	Invitrogen, Eugene, Oregon, USA	
anti-Goat IgG (H + L) Cross-Adsorbed Secondary Antibody, Alexa Fluor™ 488		donkey	1:300	Invitrogen, Eugene, Oregon, USA	
TO-PRO^R^−3 iodide 642/661			1:10,000	Molecular Probes, Eugene, Oregon, USA	
Protein Block Serum Free			Ready to use	DakoCytomation, Glostrup, Denmark	
ProLong™ Diamond Antifade Mountant			Ready to use	Invitrogen, Eugene, Oregon, USA	
Mowiol Mounting Medium			Ready to use	Società Italiana Chimici, Italy	

### Confocal immunofluorescence of muscle myenteric plexus (LMMP) whole-mount preparations

Immunofluorescence assays were performed on colonic whole-mount preparations as previously described (Antonioli et al. 2019; Caputi et al. 2017; Cerantola et al. 2022; Faggin et al. 2025a). After washing with phosphate-buffered saline (PBS), fresh isolated distal 3 cm-colonic segments were fixed in 4% paraformaldehyde (PFA) in PBS for 2 h at room temperature. Using a dissecting microscope, longitudinal muscle myenteric plexus (LMMP) whole-mount preparations were isolated and pinned down on the bottom of Sylgard-coated dishes, washed in PBT (PBS + 0.3% Triton X-100) with gentle shaking, and blocked with 5% bovine serum albumin (BSA) in PBT for 1.5 h at room temperature, as previously described (Cerantola et al. 2022; Faggin et al. 2025a). After the fixation/permeabilization procedure, colonic LMMP whole mount preparations were incubated overnight at room temperature with the following primary antibodies: anti-HuC/D, anti-mouse SP, anti-Iba-1, anti-CD64, anti-F4/80 (see Table 1). Then, all samples were washed and incubated for 2 h at room temperature with appropriate secondary antibodies (see Table 1). After three washes, LMMP whole mount preparations were mounted on glass slides using a Mowiol Mounting Medium (CitiFluor™ Mountant Solution AF1, Società Italiana Chimici, Italy) and stored in the dark at −20 °C until confocal analysis. Negative controls were obtained by incubating sections with isotype-matched control antibodies at the same concentration as the primary antibody and/or preincubating each antibody with the corresponding control peptide (final concentration as indicated by the manufacturer’s instructions). Images were acquired using a Zeiss LSM 800 confocal imaging system (Oberkoken, Germany) equipped with a 40 × objective for LMMP whole mount preparations or an oil-immersion 63 × objective (NA 1.4) for colonic cryosections. Z-series images (10 planes of 1024 × 1024 pixels for cryosections, or 20 planes of 512 × 512 pixels for LMMP whole mount preparations) were processed as maximum intensity projections. All microscope settings were kept constant for all images. In colonic LMMP preparations, the enteric neuronal (HuC/D^+^ or SP^+^) and macrophagic (CD64^+^ or F4/80^+^) populations were analyzed by counting the total number of cells in 6 randomly chosen images per mouse (*N* = 3–5 mice/group), and then normalized per myenteric ganglion area, as previously described (Cerantola et al. 2022; Faggin et al. 2025a). Immunofluorescence images were analyzed by selecting only the z-stack planes in which the myenteric ganglia were fully identifiable, based on the presence or absence of HuC/D^+^ neurons or SP^+^ neuronal fibers. Consequently, only IBA1^+^, CD64^+^, or F4/80^+^ positive macrophages located within these selected planes were considered for the analysis, allowing us to assess the spatial proximity of the macrophage population to the myenteric ganglia. All image analyses were performed using ImageJ (Fiji) Version Number 1.54i.

### Data and statistical analysis

The statistical analysis of data complies with the requirements of good laboratory practices (GLP). The statistical analysis was undertaken only for studies where each group size was at least *n* = 6 for biochemical and functional experiments and at least *n* = 3 for morphological experiments. In particular, the results are presented as mean ± standard error of the mean (S.E.M.). All the group sizes were designed to be homogeneous. n refers to the number of mice and statistical analysis was carried out using these independent values. The significance of differences was evaluated by one-way or two-way ANOVA followed by the appropriate post hoc test. Post hoc tests were conducted only if F in ANOVA (or equivalent) achieved a statistical significance lower than 0.05 and there was no significant variance in homogeneity. *P* values < 0.05 were considered representative of significant statistical differences. All statistical procedures were performed by commercial software (GraphPad Prism, version 9.0, RRID: SCR_002798, GraphPad Software Inc., San Diego, CA, USA).

## Results

### CCR2/CCL2 pathway contributes to the development of HFD-induced obesity

To investigate the role of CCR2/CCL2 in HFD-induced obesity, we carried out experiments in mice fed with HFD for 8 weeks treated with the CCR2 antagonist (RS504393), in order to inhibit CCL2/CCR2 interactions and consequently gut-homing of blood monocytes (Sayed et al. 2022; Tu et al. 2020). HFD mice displayed a significant increase in BMI, body and epididymal fat weight (Fig. 1B-D). HFD feeding also significantly increased spleen and liver weight (Fig. 1E-F), thus supporting previous findings showing that HFD-induced metabolic dysregulation and inflammation contribute to splenomegaly and liver damage, respectively (Buchan et al. 2018). HFD mice also displayed a significant increase in plasma insulin levels as compared with SD mice (Fig. 1G), confirming metabolic dysfunction and insulin resistance following HFD intake. Of note, treatment with CCR2 inhibitor to HFD mice decreased significantly BMI, body weight gain as well as epidydimal fat, spleen and liver weight as compared with vehicle-treated HFD mice (Fig. 1B-F). CCR2 antagonist also reduced insulin levels in HFD mice, restoring values comparable to SD animals (Fig. 1G). In addition, CCR2 inhibition to SD mice decreased BMI, body weight, spleen weight and insulin levels as compared with SD vehicle-treated animals (Fig. 1B-E). The decrease in body weight following RS504393 treatment could depend on the effect of CCR2 blockade on insulin sensitivity, known to regulate adipose tissue metabolism, energy homeostasis and body weight (Cignarelli et al. 2019; Sullivan et al. 2013). Of note, HFD mice exhibited increased levels of IL-1β in colonic tissue compared with vehicle-treated SD animals (Fig. 1H), mirroring an enhanced intestinal inflammatory state, consistent with the low-grade intestinal inflammation, typically observed in obese patients (Rohm et al. 2021). Treatment with the CCR2 inhibitor significantly reduced colonic IL-1β levels in HFD mice (Fig. 1H). These results suggest that obese mice treated with CCR2 antagonist were less susceptible to the development of HFD-induced obesity and related gut inflammation, thus highlighting the involvement of CCR2/CCL2 signaling in the pathophysiology of related-inflammation obesity.

### HFD-induced obesity is associated with blood monocytes homing in the enteric neuromuscular layer

**Fig. 2:**
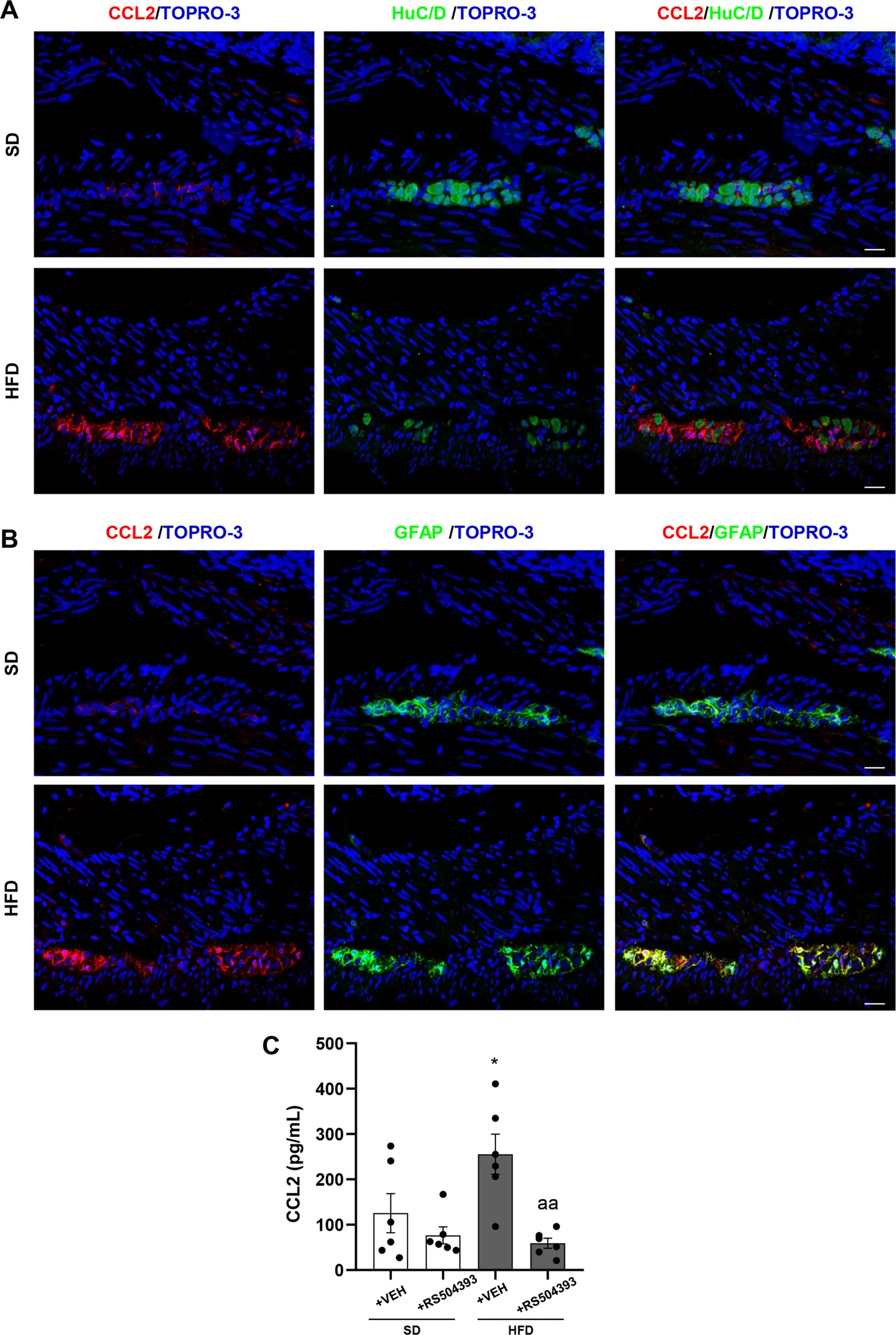
HFD induces CCL2 expression in enteric neurons and glial cells. **A** Representative confocal images of CCL2 (red)/HuC/D (green) immunofluorescence in colonic *tunica submucosa* and *muscularis* from mouse fed with SD and HFD (bars = 20 µm; *N* = 5 mice/group). **B** Representative confocal images of CCL2 (red)/GFAP (green) immunofluorescence in colonic *tunica submucosa* and *muscularis* from mouse fed with SD and HFD (bars = 20 µm; *N* = 5 mice/group). Panels show adjacent serial sections of the same myenteric ganglia from SD or HFD mice, stained for CCL2 with HuC/D or GFAP. **C** CCL2 levels in colonic tissues of SD and HFD mice treated with vehicle or RS504393. Data are expressed as mean ± S.E.M. (*N* = 6 mice/group). One-way ANOVA followed by Tukey post hoc test results: *p < 0.05 vs. vehicle-treated SD; ^aa^*P* < 0.01 vs. vehicle-treated HFD. *Abbreviations:* SD, standard diet; HFD, high-fat diet

**Fig. 3:**
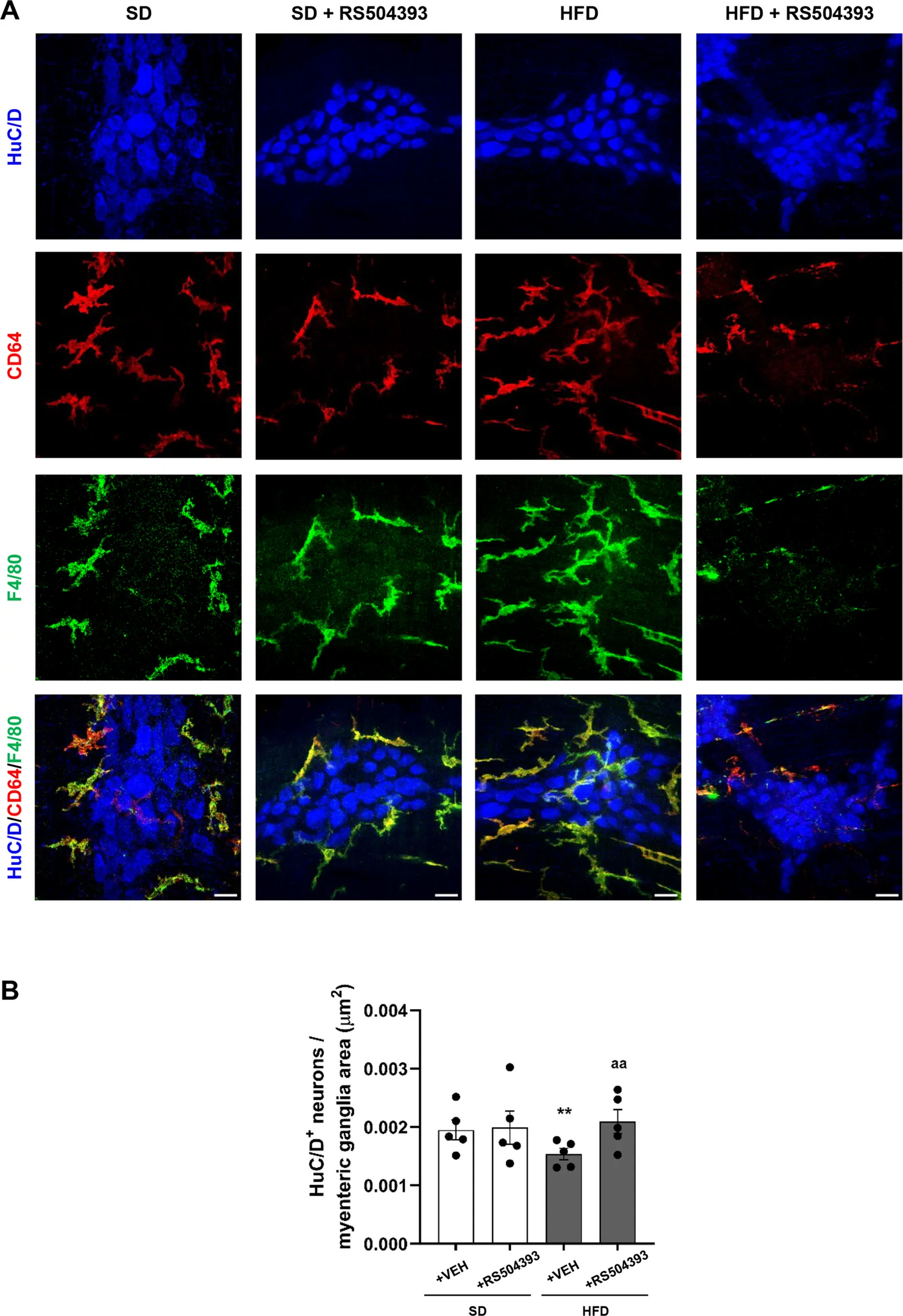
CCR2 antagonist reduces CCL2/CCR2-dependent monocyte recruitment in the enteric plexus. **A** Representative confocal microphotographs showing the localization of HuC/D^+^ neurons (blue) and the distribution of CD64^+^ (red), and F4/80^+^ (green) macrophages in longitudinal muscle–myenteric plexus (LMMP) preparations of SD or HFD mice treated with vehicle or RS504393 (bars = 22 μm; *N* = 3 mice/group). **B** Quantification of total number of HuC/D^+^ neurons performed on LMMP whole-mount preparations from SD or HFD mice treated with vehicle or RS504393. (*N* = 5 mice/group. One-way ANOVA followed by Tukey post hoc test results: **P < 0.01 vs. vehicle-treated SD; ^aa^*P* < 0.01 vs. vehicle-treated HFD. *Abbreviations:* SD, standard diet; HFD, high-fat diet; LMMP, longitudinal muscle–myenteric plexus

Several intestinal inflammatory conditions, including obesity, are characterized by an increase in pro-inflammatory macrophages, both residents (Wehner et al. 2007, 2009) and CCL2/CCR2-driven blood-derived (Farro et al. 2017), in colonic tissues that contribute to the ongoing enteric inflammatory processes and gut motor disturbances (Rohm et al. 2021; Antonioli et al. 2019; Boeckxstaens and Jonge 2009; Buscail and Deraison 2022; Stein et al. 2018). Thus, we investigated the local production and distribution of CCL2 together with the distribution of pro-inflammatory macrophages in colonic neuromuscular layer from obese mice. First, we found an increase in colonic CCL2 chemokine levels in myenteric plexus in HFD mice as compared with SD animals (Fig. 2A-C). In addition, double immunofluorescence staining of CCL2 with GFAP or HuC/D, markers of enteric glial and neurons, respectively, showed an increase of CCL2 immunopositivity in GFAP^+^ myenteric glia from HFD mice as compared with SD (Fig. 2A-B). These findings indicate an involvement of enteric glia and neurons in CCL2 release, that, in turn, could contribute to monocyte gut-homing (Isidro and Appleyard 2016). Indeed, in subsequent immunodetection experiments were carried out on colonic whole-mount preparations for CD64 and F4/80 antigens, regarded as colonic mature macrophages markers highly expressed by 72–96 h post gut homing (Isidro and Appleyard 2016). Our results showed a high co-localization of CD64^+^/F4/80^+^ macrophages in close proximity of myenteric plexus (Fig. 3A) from colonic tissues of HFD animals, suggesting an increase in activated macrophages within the enteric neuromuscular layer from obese mice. Such an increase can result from the polarization towards pro-inflammatory phenotype of both CCR2/CCL2-dependent recruited and resident macrophages. Indeed, it is noteworthy that the CD64^+^/F4/80^+^ macrophage maturation also depends on the activation of enteric CCR2/CCL2 pathway (Isidro and Appleyard 2016).

**Fig. 4:**
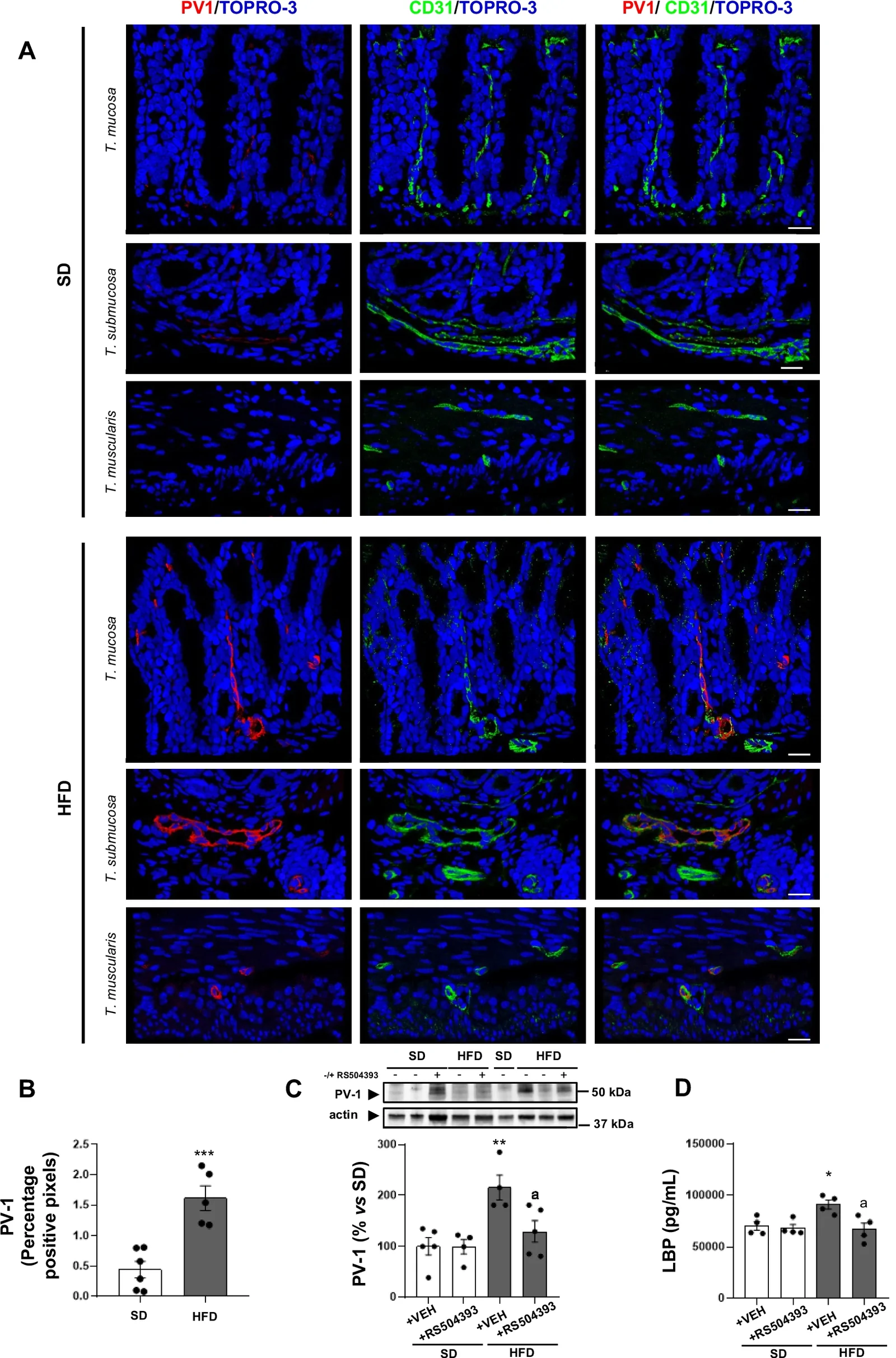
CCR2 antagonist preserves intestinal vascular integrity and restores intestinal permeability. **A** Representative confocal images of PV1 (red)/CD31 (green) immunofluorescence in colonic *tunica mucosa*, *submucosa* and *muscularis* from mouse fed with SD and HFD (bars = 20 µm; *N* = 3–5 mice/group). **B** Column graph represents the mean of percentage of positive pixels (PPP) ± SEM of PV1 analysis. **C** Densitometric analysis of PV-1 protein levels by Western blot with representative immunoblot in colon tissues of SD or HFD mice treated with vehicle or RS504393. **D** Lipopolysaccharide-binding protein (LBP) levels in plasma of SD or HFD mice treated with vehicle or RS504393 (*N* = 4–5 mice/group). One-way ANOVA followed by Tukey post hoc test results: *p < 0.05, ***p < 0.001 vs. vehicle-treated SD; ^a^*P* < 0.051 vs. vehicle-treated HFD *Abbreviations:* SD, standard diet; HFD, high fat diet

Supporting these results, in colonic vessels of obese mice we detected also a switch of the endothelium phenotype towards an activated form characterized by an increase of both P-selectin (Additional Fig. 1A) and PV-1 expression (Fig. 4A-C) together with an increased levels of plasma LBP (Fig. 4D), suggesting an impairment of the gut vascular barrier and consequently of its permeability.

### CCR2/CCL2 signaling contributes to colonic dysmotility associated with obesity, through the modulation of enteric tachykininergic motor pathways

**Fig. 5:**
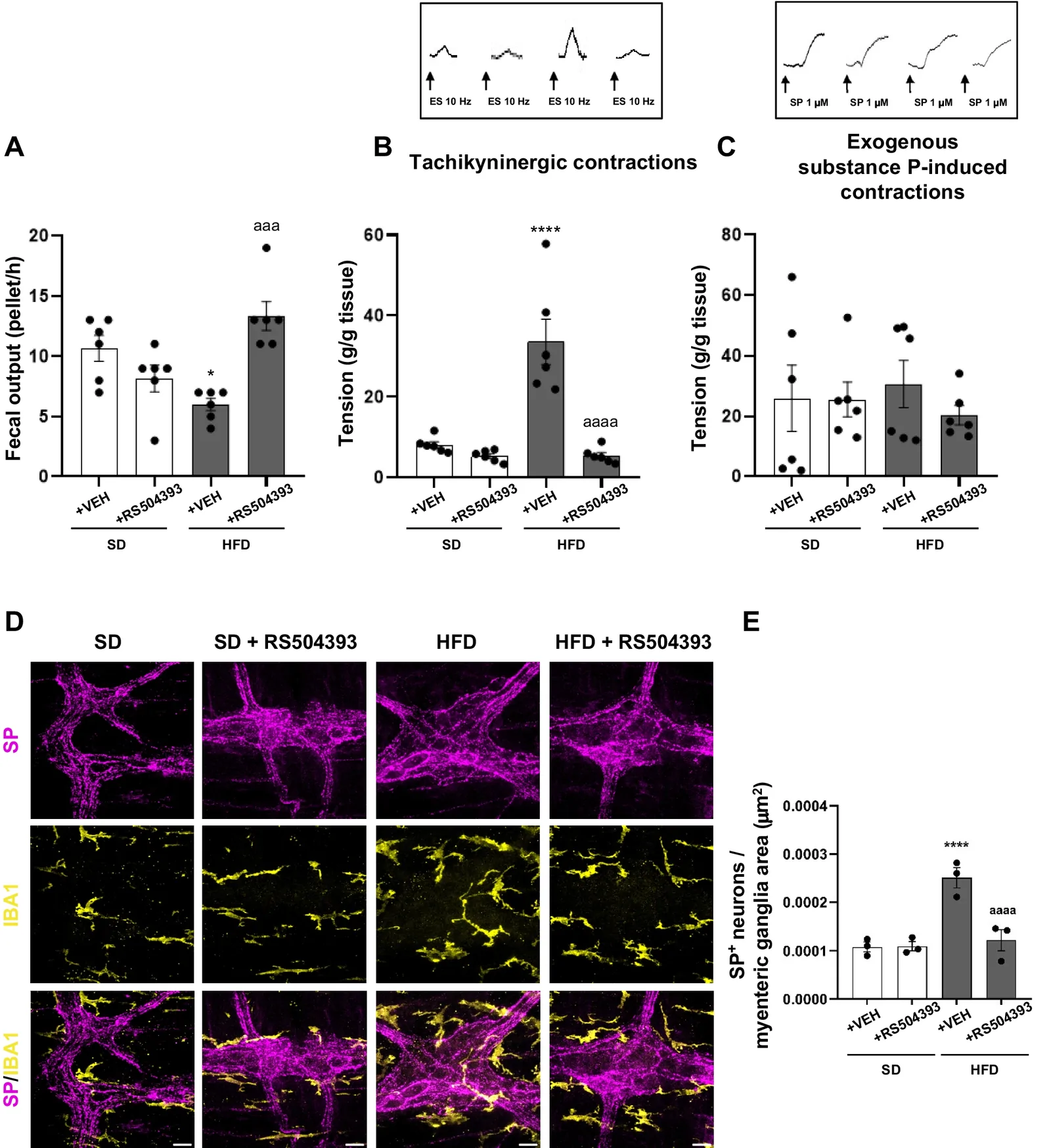
CCR2 antagonist restores colonic dysmotility associated with obesity, through the modulation of enteric tachykininergic motor pathways. **A** Fecal output expressed as number of pellets in 1 h in SD and HFD mice treated with vehicle or RS504393. (*N* = 6 mice/group). **B** NK_1_-mediated tachykininergic contractions of colonic longitudinal smooth muscle preparations isolated from SD and HFD mice treated with vehicle or RS504393 in Krebs solution containing L-NAME (nitric oxide synthase inhibitor, 100 μM), guanethidine (adrenergic blocker, 10 μM), atropine (muscarinic receptor antagonist, 1 μM), GR159897 (NK2 receptor antagonist, 1 μM), and SB218795 (NK3 receptor antagonist, 1 μM) (*N* = 6 mice/group). **C** Contractions evoked by exogenous SP (1 μM) in colonic preparations of SD and HFD mice treated with vehicle or RS504393 in Krebs solution containing L-NAME (100 μM), guanethidine (10 μM), atropine (1 μM), GR159897 (1 μM), and SB218795 (1 μM), in the presence or absence of L-732,138 (NK1 receptor antagonist, 10 μM) or tetrodotoxin (TTX, 1 μM) (*N* = 6 mice/group). Tracings in the inset on the top of panels display the contractile responses per each experimental group, indicated by an arrow. One-way ANOVA followed by Tukey post hoc test results; ****p < 0.0001 vs. vehicle-treated SD; ^aaaa^*P* < 0.0001 vs. vehicle-treated HFD. **D** Representative confocal microphotographs of dual-label immunohistochemistry for SP^+^ neuronal fibers (magenta) and Iba1^+^ (yellow) macrophages in LMMP preparations of SD or HFD mice treated with vehicle or RS504393 (bars = 22 μm). **E** Quantification of total number of SP^+^ neurons in LMMP preparations of SD or HFD mice treated with vehicle or RS504393 (*N* = 3 mice/group). One-way ANOVA followed by Tukey post hoc test results: *p < 0.05 ****P < 0.0001 vs vehicle-treated SD; ^aaa^*P* < 0.001, ^aaaa^*P* < 0.0001 vs. vehicle-treated HFD. *Abbreviations:* SD, standard diet; HFD, high-fat diet; SP, substance P; LMMP, longitudinal muscle–myenteric plexus

In this set of experiments, the role of CCR2/CCL2 signaling in colonic dysmotility from obese mice was assessed. HFD mice were characterized by a significant decrease in stool frequency, as compared with SD mice (Fig. 5A), confirming previous studies showing that HFD-induced obesity is associated with a decrease in colonic transit (Pellegrini et al. 2021). Of note, treatment with CCR2 antagonist to HFD animals was associated with significant decrease of CCL2 levels (Fig. 2C) along with a recovery of in vivo colonic motility as compared with HFD mice (Fig. 5A), while no changes were detected in CCR2 antagonist-treated SD animals (Fig. 5A), thus suggesting that the activation of CCR2/CCL2 pathway in obesity contributes to colonic dysmotility associated with obesity.

Then, we went on to examine the role of CCR2 in the alterations of enteric tachykinergic neurotransmission in colonic tissues from obese mice, since the enteric SP/NK1 neuronal pathway undergoes significant rearrangements in obesity, contributing to colonic motor dysfunctions (D’Antongiovanni et al. 2020a).

During equilibration, distal colonic preparations displayed spontaneous rapid contractions, which were low in amplitude and generally stable throughout the experiment. No significant differences in spontaneous motor contractions were detected among vehicle-treated or CCR2 inhibitor-treated SD mice (mean amplitude: 10.7- and 11.7-g/g tissue, respectively) to among vehicle-treated or CCR2 inhibitor-treated HFD animals (mean amplitude 10.2- and 11.7-g/g tissue, respectively). Electrically evoked responses were characterized by phasic contractions followed, in some cases, by after-contractions of variable amplitude. In colonic longitudinal muscle preparations maintained in Krebs solution containing L-NAME, guanethidine, GR159897, SB218795 and atropine, the application of ES was associated with contractile responses, almost completely abolished by L-732,138 or tetrodotoxin, indicating the recruitment of postganglionic tachykininergic motor neurons (Additional Fig. 1B-C). Of note, the electrically evoked TTX-insensitive colonic contractions can depend on the activation of TTX-resistant Na channels, TTX-resistant purinergic pathways in enteric neurons and glia cells or TTX-insensitive glial stimulation (Beyak et al. 2004; Copel et al. 2013; Sage et al. 2007; Padilla et al. 2007; Rugiero et al. 2003; Gulbransen and Sharkey 2009, 2012; Brown et al. 2016).

Under these conditions, electrically evoked NK_1_-mediated tachykininergic colonic motor responses were significantly enhanced in HFD animals, as compared with SD animals (Fig. 5B), while no changes were detected in the myogenic motor responses evoked by exogenous SP plus tetrodotoxin (Fig. 5C). Of interest, HFD mice treated with CCR2 antagonist displayed a significant decrease in electrically evoked NK_1_-mediated tachykininergic contractions as compared with vehicle-treated HFD mice (Fig. 5B). Of note, electrically evoked tachykininergic colonic contractions both in SD and HFD conditions can depend on intrinsic tachykininergic motor neurons, extrinsic afferents nerves and SP interneurons (Bartho et al. 2008). No difference was observed in the contractions induced by exogenous SP (10 µM) in the presence of tetrodotoxin (1 µM), among all experimental groups (Fig. 5C). These results indicate that CCR2 contributes to colonic dysmotility associated with obesity, through the modulation of enteric tachykininergic neurotransmission.

Subsequently, we examined whether the above motor changes could depend on variations of the density of enteric tachykininergic neurons and fibers. This goal was pursued by immunofluorescence analysis, which revealed that HFD-induced obesity is associated with an increased SP expression in myenteric neurons and fibers, while the density of HuC/D-stained neurons was significantly decreased (Figs. 3C and 5E), thus suggesting a rearrangement of the enteric tachykininergic neuronal coding, which could contribute to the enhancement of the evoked colonic neurogenic tachykininergic contractions, as shown in our functional experiments.

**Fig. 6:**
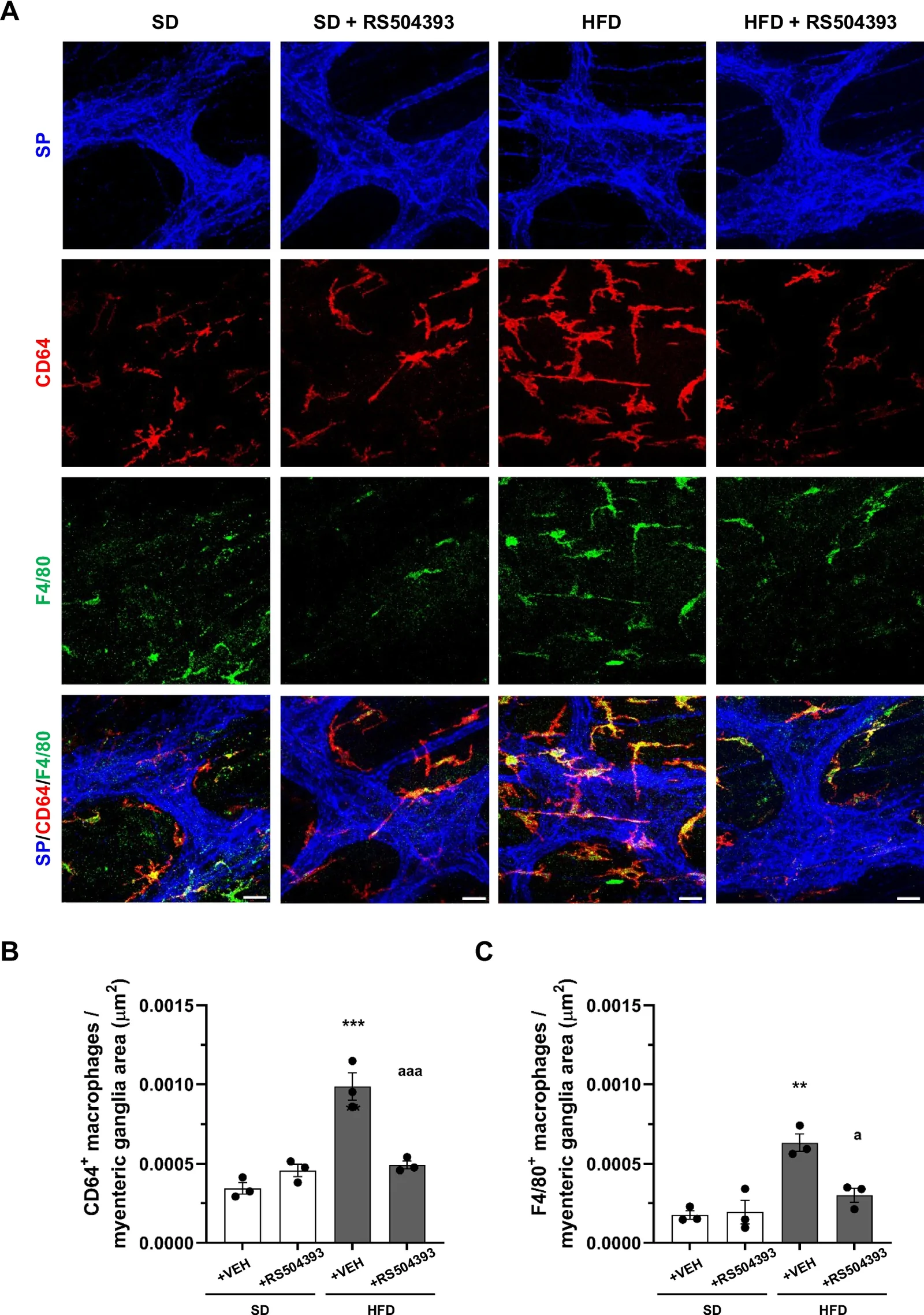
CCR2 antagonist reduces CCL2/CCR2-dependent monocyte recruitment in proximity to substance P–positive enteric neurons and fibers. **A** Representative confocal microphotographs showing the localization of SP + (blue) neuronal bodies and fibers and the distribution of CD64^+^ (red) and F4/80^+^ (green) macrophages in LMMP preparations, obtained from SD or HFD mice treated with vehicle or RS504393 (bars = 22 μm). (*N* = 3 mice/group). **B**-**C** Quantification of total number of CD64^+^ or F4/80^+^ macrophages in LMMP preparations, obtained from standard diet (SD) or high fat diet (HFD) mice treated with vehicle (VEH) or RS504393. *N* = 3 mice/group. One-way ANOVA followed by Tukey's post hoc test results. **P < 0.01 ***P < 0.001 vs vehicle-treated SD; a*P* < 0.05, aaa*P* < 0.001 vs. vehicle-treated HFD. Abbreviations: SD, standard diet; HFD, high-fat diet; SP, substance P; LMMP, longitudinal muscle–myenteric plexus

Of interest, in the colonic myenteric plexus of HFD mice, we showed an increase of SP immunopositivity along with an increase in the number of Iba1^+^ (pan macrophage marker, (Yip et al. 2024)) macrophages in proximity to the myenteric SP^+^ neurons and fibers (Fig. 5D-E), confirming an increase in activated macrophages in the myenteric plexus in HFD-fed mice. In addition, CCR2 blockade was associated with a reduction of SP immunopositivity along with a decrease in the number of Iba1^+^ and CD64^+^/F4/80^+^ macrophage in proximity to the myenteric SP^+^ fibers, while it did not affect the decreased number of HuC/D^+^ myenteric neurons (Figs. 5D-E, 6A-C and 3B), thus indicating the involvement of CCR2-dependent blood monocyte recruitment in ENS neuroplasticity associated with obesity. Supporting these results, we observed, by triple-immunofluorescence analysis, the presence of Iba-1^+^ macrophages in close proximity to myenteric blood vessels, identified by the endothelial marker CD31, and SP^+^ tachykininergic fibers in HFD colonic muscle (Additional Fig. 2B).

Overall, these results suggest that, in the presence of obesity, an overactivation of CCR2/CCL2 signaling, promotes the blood monocyte recruitment in the enteric neuromuscular compartment, which, polarizing towards mature macrophage phenotype, contributes to colonic dysmotility through the direct modulation of enteric tachykininergic neurotransmission.

## Discussion

Obesity-related inflammation has been directly linked to the development of the most serious comorbidities associated with this condition, including GI dysfunctions. In this context, the occurrence of enteric immune/inflammatory responses (Rohm et al. 2021), following gut barrier impairment and pathogen translocation, has been found to contribute to the pathophysiology of obesity and to directly influence enteric neuronal circuits, with consequent intestinal motor dysfunctions (D’Antongiovanni et al. 2023, 2025). In particular, previous studies demonstrated that activation of enteric NLRP3 inflammasome in macrophages contributed to colonic dysmotility in HFD-induced obese mice by altering excitatory tachykininergic neurotransmission, whereas depletion of intestinal macrophages or NLRP3 gene deletion restored colonic motor functions (Pellegrini et al. 2021; Antonioli et al. 2019). In this context, both intestinal immune/inflammatory cells and enteric neurons respond to mucosal inflammation by facilitating the homing of blood monocytes into the gut in a CCR2-dependent manner (Baassiri et al. 2024). Indeed, a recent study reported that obese patients displayed gut inflammation characterized by accumulation of pro-inflammatory intestinal macrophages, potentially derived from recruited blood monocytes (Rohm et al. 2021). However, in the setting of obesity, whether gut infiltration of blood monocytes, besides occurring within the tunica mucosa, can (i) reach the neuromuscular layers, (ii) polarize toward a mature pro-inflammatory muscularis macrophage phenotype, and (iii) contribute to gut motor dysfunctions remains to be investigated.

Based on the above background, the present study was designed to elucidate the role played by gut-homing monocytes in bowel dysmotility in a murine model of HFD-induced obesity. In particular, based on our previous evidence showing the interplay between enteric inflammation and SP/NK1 neuronal pathways in colonic dysmotility associated with obesity, we focused on CCR2/CCL2-dependent monocyte recruitment into the enteric neuromuscular compartment of obese mice and on its effects on alterations in colonic tachykininergic neurotransmission.

Taken together, our results provide novel and intriguing evidence regarding the role of gut-homing monocytes in the remodelling of enteric tachykininergic neuronal coding in HFD-fed animals. In particular, the present study highlights the following novel findings: (1) CCR2/CCL2 signalling is involved in several pathophysiological processes associated with obesity, including body weight gain, splenomegaly, and enteric inflammation; (2) CCR2/CCL2-dependent gut-homing blood monocytes polarize towards a mature pro-inflammatory muscularis macrophage phenotype within the enteric neuromuscular compartment; and (3) CCR2-dependent monocyte trafficking contributes to enteric dysmotility associated with obesity through the direct modulation of enteric tachykininergic pathways.

As previously reported, HFD intake to WT mice was associated with a significant increase in body weight, epididymal fat weight, BMI, spleen and liver weight as well as in insulin levels along with the occurrence enteric inflammation, confirming that HFD exposure is associated with the development of obesity and related gut inflammation (Antonioli et al. 2017, 2019; Buchan et al. 2018). Of interest, such HFD-dependent effects were not detected in obese mice treated with a CCR2 antagonist, corroborating previous preclinical evidence showing a pivotal role of CCR2 in the pathophysiology of obesity and related inflammation and comorbidities (Correction: CCR2-dependent monocyte-derived macrophages resolve inflammation and restore gut motility in postoperative ileus 2019; Sullivan et al. 2013; Weisberg et al. 2006; Kim et al. 2016; Kang et al. 2010; Huh et al. 2018; Tamura et al. 2008; Velasquez-Mieyer et al. 2003).

Subsequently, we focused on the recruitment of blood monocytes in enteric neuromuscular layer in obese mice and we found increase in the number of Iba-1^+^/CD64^+^/F4/80^+^ macrophages, regarded as both pro-inflammatory resident and recruited macrophages (including *muscularis*) (Isidro and Appleyard 2016; Dora et al. 2021), in close spatial association with the myenteric plexus of HFD-fed mice. These results are in line with a recent study showing an increase in the number of CD14^+^ (the human homologue of mouse CD64) pro-inflammatory macrophages in colonic tissues from obese patients concomitantly with elevated intermediate blood monocytes (Rohm et al. 2021). Of interest, in inflamed murine gut tissues, the accumulation of monocyte-derived CD64^+^/F4/80^+^ mature macrophages depends on activation of the enteric CCR2/CCL2 pathway (Farro et al. 2017; Isidro and Appleyard 2016; Kurihara et al. 1997; Zigmond et al. 2012). Accordingly, we observed increased CCL2 levels in enteric neurons and glial cells, regarded as a source of CCL2 during enteric inflammation that drives the trafficking of CCR2 + blood monocytes into inflamed intestinal tissues (Hatoum et al. 2003). In this context, since monocyte gut homing is further facilitated by the increased gut vascular barrier permeability and endothelial adhesion molecule expression (Deban et al. 2008; Hatoum et al. 2003), we observed that HFD mice displayed an increased expression of P-selectin and PV-1 in colonic tissues, together with elevated plasma LBP levels, thus supporting the occurrence of intestinal barrier dysfunction and endothelial activation. These finding suggest that, increased colonic CCL2 release from enteric neurons and glia, together with the increase in endothelial adhesion molecule expression and gut vascular permeability drive and facilitate the recruitment of blood monocytes into the enteric tunica muscularis in obesity, that, in turn, polarize towards to mature pro-inflammatory macrophages. Supporting these results, CCL2, besides exerting chemotactic activity, has been found to influence endothelial hyperpermeability by altering tight junction-associated proteins in brain microvascular endothelial cells during central nervous system inflammation, with consequent impairment of blood–brain barrier integrity (Stamatovic et al. 2006).

Several studies have reported that muscularis macrophages control gut motility under physiological conditions and actively participate in the onset of bowel motor dysfunctions during intestinal inflammation associated with several disorders, including obesity (Antonioli et al. 2019; Muller et al. 2014; Choi et al. 2024). Indeed, depletion of abdominal macrophages in HFD-fed mice, besides reducing colonic inflammation, was also associated with recovery of the altered tachykininergic contractions observed in obese animals (Antonioli et al. 2019). Based on this evidence, we went to explore the specific role of monocyte-derived muscularis macrophages in HFD-induced colonic dysmotility. In particular, we designed specific sets of experiments in HFD-fed mice treated with a selective CCR2 antagonist. Under these experimental conditions, CCR2 receptor blockade, besides decreasing colonic CCL2 levels and restoring gut barrier integrity, normalized the morphofunctional alterations of colonic tachykininergic pathways, with consequent recovery of in vivo colonic transit. Indeed, increased colonic SP-mediated tachykininergic contractions have been implicated in altered peristaltic movements and consequent impairment of colonic propulsive activity (Bornstein et al. 2004). Therefore, restoration of in vivo colonic transit following blockade of CCR2-dependent gut-homing monocytes could result from the normalization of colonic tachykininergic contractile responses, which were significantly increased following HFD exposure. Of note, colonic motility arises from complex interactions between smooth muscle layers and excitatory/inhibitory neurotransmitter pathways. Thus, the neuronal loss observed in HFD-fed mice could also involve other excitatory and/or inhibitory neuronal popuilations, including cholinergic and nitrergic pathways, with consequent colonic dysmotility (Faggin 2025b). In addition, CCL2 released by enteric glia may further contribute to enteric dysmotility in HFD-fed mice. Indeed, in a previous study, selective depletion of enteric glia with fluorocitrate normalized electrically evoked contractions in obese mice (Antonioli et al. 2020; D’Antongiovanni et al. 2025).

Taken together, these results provide convincing evidence that, in the setting of obesity, CCR2- driven blood monocytes recruitment directly impair enteric tachykininergic neurotransmission highlighting the relevance of enteric neuro-immune interplay in the colonic dysmotility associated with obesity. Such an effect can be ascribed to the direct inhibition of CCR2-dependent monocyte gut homing rather than to secondary effects associated with improvement of the obese condition, as demonstrated by previous studies in which depletion of intestinal macrophages directly improved colonic motility in HFD-fed mice (Antonioli et al. 2019). However, besides circulating monocyte-derived macrophages, the contribution of resident muscularis macrophages to bowel dysmotility in the setting of obesity remains to be clarified and could represent the logical continuation of this line of research.

Of interest, a previous study revealed the existence of a link between CCR2/CCL2 signaling and SP that contributes to nociceptor sensitization and peripheral inflammation (Dansereau et al. 2021). In particular, activation of CCR2/CCL2 following painful peripheral inflammatory stimuli increased SP release in dorsal root ganglia (DRG) contributing to pain sensitization and peripheral inflammation. Indeed, CCR2 blockade reduced mRNA expression of CCL2 and SP, as well as peripheral SP release, with consequent reduction of neurogenic inflammation and hind paw edema (Dansereau et al. 2021). Accordingly, our experiments showed that CCR2 inhibition in HFD-fed mice reduced the number of recruited CD64^+^/F4/80^+^ macrophages in proximity to myenteric SP neuronal cells and fibers, supporting the existence of a direct link between CCR2/CCL2 and SP in sustaining enteric inflammation and colonic dysmotility in obesity.

The present study has some limitations, including the lack of direct mechanistic evidence of the enteric neuroimmune cell interactions in the onset of colonic dysmotility associated with obesity. In addition, only male mice were included in the experimental design. In this context, since sex-related differences in immune responses and metabolic alterations have been described in obesity, future studies with both male and female mice would allow to better discriminate sex-related neuroimmune responses.

## Conclusions

In conclusion, the present study shows that CCR2/CCL2/SP signaling regulates the interplay between gut homing of innate immune/inflammatory cells and enteric tachykininergic neuronal pathways, thereby playing a relevant role in the enteric motor dysfunctions associated with obesity. Based on this knowledge, the CCR2/CCL2 pathway may represent a suitable molecular target for the development of novel pharmacological entities for the treatment of enteric inflammation and motor dysfunctions.

## Supplementary Information

Additional file 1.

## Data Availability

The datasets used and/or analysed during the current study are available from the corresponding author on reasonable request.

## Abbreviations

BMI: Body Mass Index
CCL2: C–C Motif Chemokine Ligand 2 (Monocyte Chemoattractant Protein-1, MCP-1)
CCR2: C–C Motif Chemokine Receptor 2
CD62P: P-selectin
GFAP: Glial Fibrillary Acidic Protein
GI: Gastrointestinal
HFD: High-Fat Diet
HuC/D: Neuronal RNA-binding proteins HuC and HuD
L-NAME: NG-Nitro-L-arginine Methyl Ester (NOS inhibitor)
LBP: Lipopolysaccharide-Binding Protein
LMMP: Longitudinal Muscle–Myenteric Plexus
NASH: Non-Alcoholic Steatohepatitis
NK1: Neurokinin-1 Receptor
NK2: Neurokinin-2 Receptor
PV-1: Plasmalemma Vesicle-Associated Protein-1
SD: Standard Diet
SP: Substance P
TOPRO-3: TO-PRO-3 Iodide (nuclear dye)
TTX: Tetrodotoxin
