# Supplementary material for: CCR2-dependent monocytes gut-homing contributes to enteric neuroplastic changes in a mouse model of diet-induced obesity
Source: Mol Med. 2026 Jul 15;32:179. doi: 10.1186/s10020-026-01560-y (PMC13651239; doi:10.1186/s10020-026-01560-y)
Supplement: Supplementary file 1 [file 10020_2026_1560_MOESM1_ESM.pdf]

Additional Figure 1

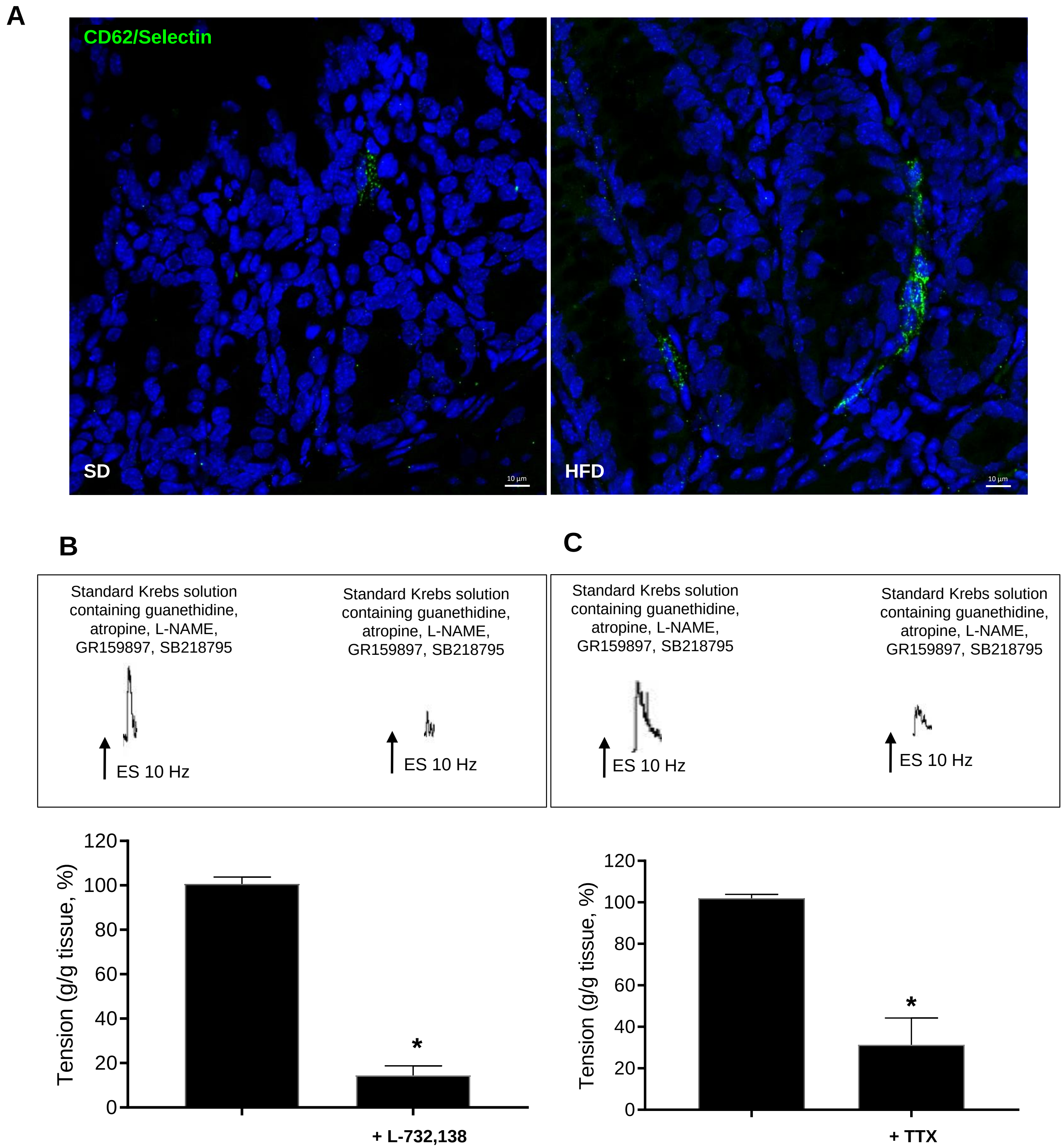

Additional figure 1. **(A)** Representative confocal images of CD62/Selectin (green) immunofluorescence in colonic mucosa from SD and HFD mice (N=4 animals). (**B-C)** Longitudinal smooth muscle preparations of distal colon maintained in Krebs solution containing L-NAME (100 μM), guanethidine (10 μM), atropine (1 μM), GR159897 (NK2 receptor antagonists, 1 μM), and SB218795 (NK3 receptor antagonists, 1 μM) with or without L-732,138 (10 mM) **(B)** or tetrodotoxin (1 mM) **(C)**, and contractile responses were elicited by electrical stimulation (ES: 0.5 ms, 30 mA, 10 s, 10 Hz). Each column represents the mean±SEM (N=4 animals)

Additional Figure 2

A

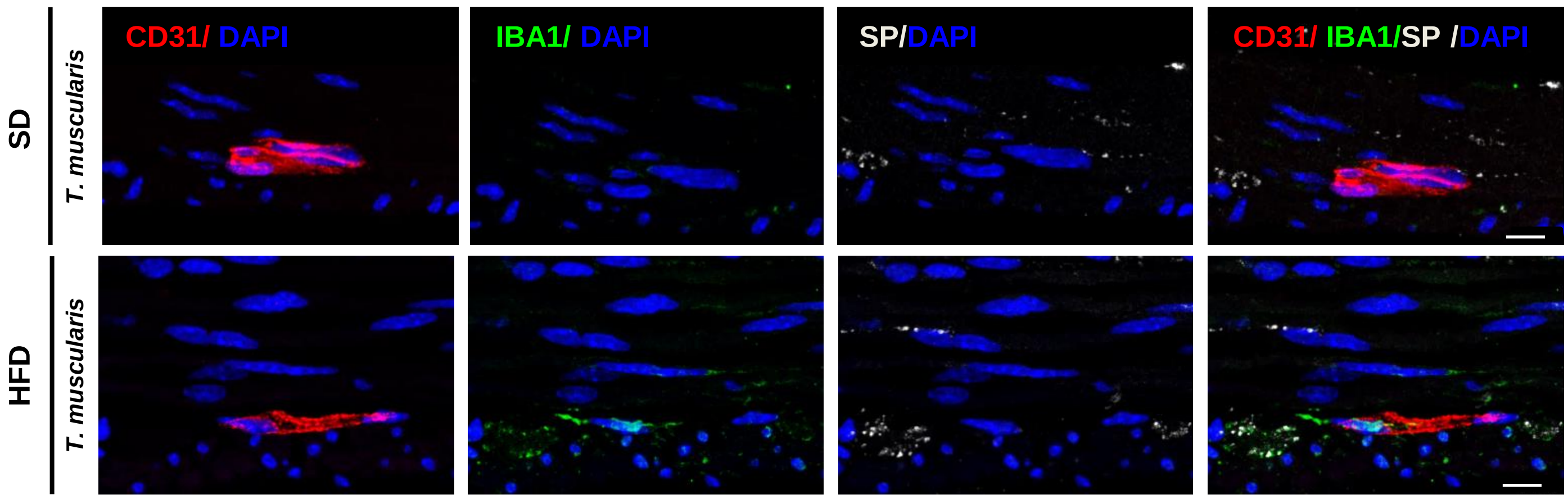

Additional figure 2. (A) Representative confocal images of CD31 (red) /Iba1 (green) / SP (gray) / DAPI (blue) multiple immunofluorescences in colonic tunica muscularis from SD and HFD mouse (N = 3 mice/group). Abbreviations: SD, standard diet; HFD, high fat diet; SP, substance P.
